# A Case of Potts’ Disease

**Published:** 1902-03

**Authors:** 


### A Case of Potts' Disease.

In a recent number of the *Brooklyn Medical Journal* Dr. W. C. Woods recites the history of an interesting case of Potts' disease. The patient, a delicate female child, aged four, became ill several years ago with the symptoms of acute Potts' disease. So severe were the symptoms that she could not wear a brace, and consequently was treated for one year by recumbency, as suggested by Dr. Mosher. In spite of this, however, a large abscess developed, which occupied about one-third of the abdominal cavity. At the same time her temperature ran from 103 to 104. Being a firm believer in the wisdom of non-interference, Dr. Wood made no effort to open the abscess, which after a time ruptured into the intestine, giving a large discharge of foul-smelling pus. Three times in two years similar abscesses formed and each time ruptured into the intestine. The child has apparently made a perfect recovery except for a slight kyphosis. For two years she has seemed to be entirely well. She plays as vigorously as any of her associates and does not even have and wear a brace. R.

